# Comparison of the terrestrial cyanobacterium *Leptolyngbya* sp. NIES-2104 and the freshwater *Leptolyngbya boryana* PCC 6306 genomes

**DOI:** 10.1093/dnares/dsv022

**Published:** 2015-10-21

**Authors:** Yohei Shimura, Yuu Hirose, Naomi Misawa, Yasunori Osana, Hiroshi Katoh, Haruyo Yamaguchi, Masanobu Kawachi

**Affiliations:** 1 Center for Environmental Biology and Ecosystem Studies, National Institute for Environmental Studies, Tsukuba, Ibaraki 305-8506, Japan; 2 Department of Environmental and Life Sciences/Electronics-Inspired Interdisciplinary Research Institute (EIIRIS), Toyohashi University of Technology, Toyohashi, Aichi 441-8580, Japan; 3 Department of Electrical and Electronics Engineering, University of Ryukyus, Nishihara, Okinawa 903-0213, Japan; 4 Life Science Research Center, Mie University, Tsu, Mie 514-8507, Japan

**Keywords:** comparative genomics, cyanobacteria, genome sequence, nitrogen fixation

## Abstract

The cyanobacterial genus *Leptolyngbya* is widely distributed throughout terrestrial environments and freshwater. Because environmental factors, such as oxygen level, available water content, and light intensity, vary between soil surface and water bodies, terrestrial *Leptolyngbya* should have genomic differences with freshwater species to adapt to a land habitat. To study the genomic features of *Leptolyngbya* species, we determined the complete genome sequence of the terrestrial strain *Leptolyngbya* sp. NIES-2104 and compared it with that of the near-complete sequence of the freshwater *Leptolyngbya boryana* PCC 6306. The greatest differences between these two strains were the presence or absence of a nitrogen fixation gene cluster for anaerobic nitrogen fixation and several genes for tetrapyrrole synthesis, which can operate under micro-oxic conditions. These differences might reflect differences in oxygen levels where these strains live. Both strains have the genes for trehalose biosynthesis, but only *Leptolyngbya* sp. NIES-2104 has genetic capacity to produce a mycosporine-like amino acid, mycosporine-glycine. Mycosporine-glycine has an antioxidant action, which may contribute to adaptation to terrestrial conditions. These features of the genomes yielded additional insights into the classification and physiological characteristics of these strains.

## 1. Introduction

Oxygen-evolving photosynthetic bacteria, known as cyanobacteria, are widely distributed throughout freshwater, seawater, brackish water, and soil surfaces, and can even be found in extreme environments, such as hot springs or polar regions.^1,2^ In addition to their ecological significance as a primary producer, cyanobacteria are well known among all photosynthetic eukaryotes as organisms that participated in the origin of plastids.^3^ Therefore, comparative genomics between cyanobacteria and photosynthetic eukaryotes has attracted significant attention.^4,5^ Genomic data are rapidly accumulating as high-throughput genome sequencers have become more prevalent in the past decade; to date, over 100 cyanobacterial genome sequences have been deposited in public databases. By comparing these genome sequences, we can discover meaningful genetic features of a given genus or species of interest.^6,7^

**Figure 1.**
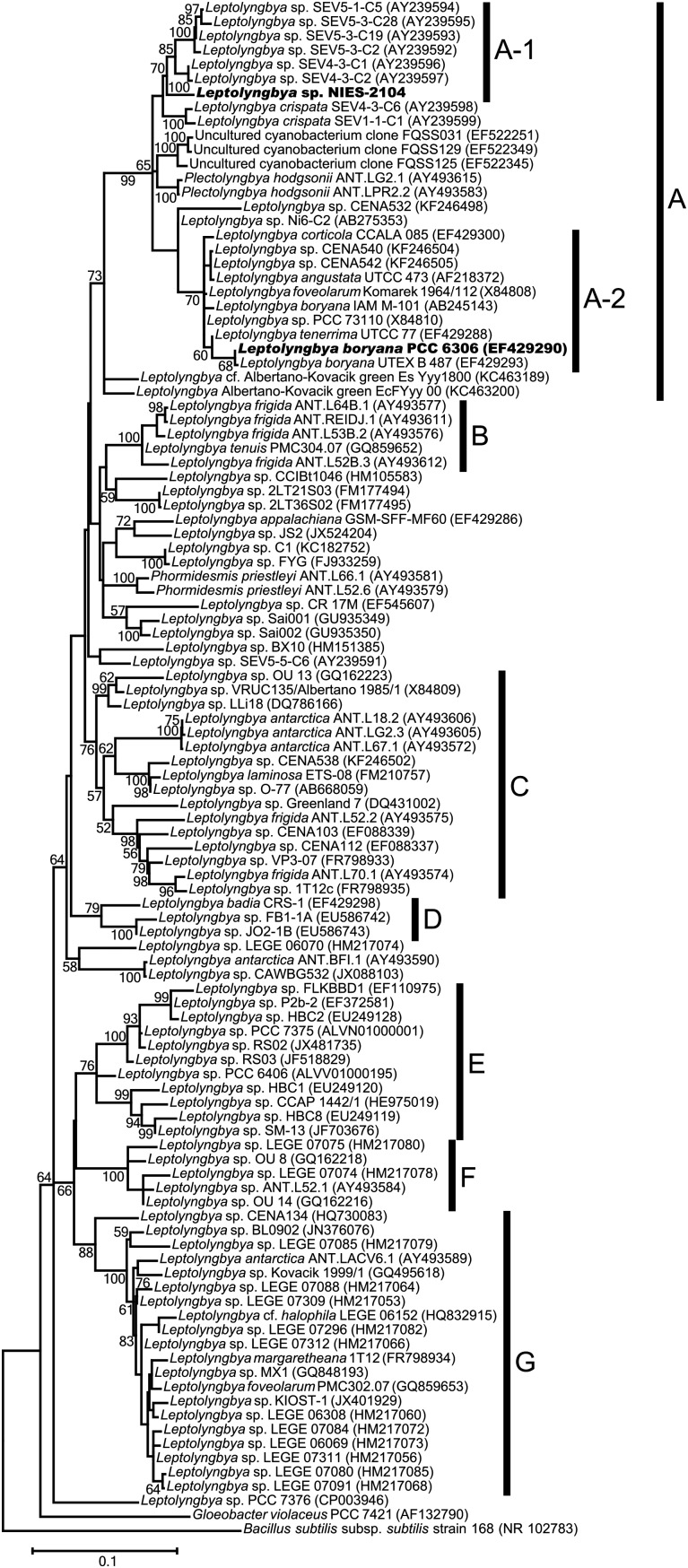
A molecular phylogenetic tree of the form genus *Leptolyngbya* based on 16S rDNA sequences. Molecular phylogenetic relationships of strains classified as *Leptolyngbya* were inferred using the maximum-likelihood method. The length of the scale bar indicates 0.1 substitutions per site. The percentages of bootstrap support of branches (>50%) are indicated at each node. Clades A–G have boot strap supports >70%. Some of these clades are at least somewhat connected to a particular environment. Clade A is an authentic *Leptolyngbya* clade, which contains the type species *Leptolyngbya boryana*. Clade A-1 is a terrestrial *Leptolyngbya* clade, which contains *Leptolyngbya* sp. NIES-2104. Clade A-2 is a freshwater *Leptolyngbya* clade, which contains *Leptolyngbya boryana* PCC 6306. A total of four of the five strains in Clade B were isolated from the Antarctic. Strains in Clade E are marine strains, except for *Leptolyngbya* sp. PCC 6406, which was isolated from freshwater, and *Leptolyngbya* sp. SM-13, which was isolated from soil.

Cyanobacteria that grow on soil surfaces are morphologically and phylogenetically divergent.^8^ The genus *Leptolyngbya*, which are thin filamentous cyanobacteria characterized by the narrow width of their cylindrical trichomes (0.5–3 µm), have been isolated from various environments, including soil surfaces. The genus *Leptolyngbya* was proposed based on morphological observations,^9^ so *Leptolyngbya* is a form genus, and the molecular phylogenetic heterogeneity of *Leptolyngbya* has been occasionally debated.^2,10,11^ Herein, the genome of an axenic cyanobacterial strain *Leptolyngbya* sp. NIES-2104, which is a terrestrial strain isolated from crusts of another terrestrial cyanobacterium, *Nostoc commune* HK-02 (NIES-2114),^12^ was analysed. *Leptolyngbya boryana*, a species that is phylogenetically related to *Leptolyngbya* sp. NIES-2104 (see Fig. 1), has been studied in detail to characterize its physiological properties, e.g. anaerobic nitrogen fixation^13^ and chlorophyll biosynthesis,^14–20^ and the near-complete genome sequence of a strain PCC 6306 was recently published.^4^ Despite the morphological similarities between *Leptolyngbya* sp. NIES-2104 and *L. boryana* PCC 6306, the habitats in which each was found were significantly different—PCC 6306 was collected from freshwater and NIES-2104 was collected from a terrestrial sample. Many environmental factors, such as oxygen level, available water supply, and the intensity and spectrum of light, can vary between water bodies and soil surfaces. To obtain insights about the evolutionary history and adaptation to particular niches by these two *Leptolyngbya* strains, we obtained the complete genome sequence of *Leptolyngbya* sp. NIES-2104 and compared it with that of *L. boryana* PCC 6306. We focused specifically on genes that are likely associated with their niche differentiation, that are genes operating under micro-oxic conditions (genes for anaerobic nitrogen fixation and micro-oxic condition-inducible tetrapyrrole biosynthetic genes); genes that are associated with desiccation tolerance (genes for trehalose biosynthesis); and genes for ultraviolet (UV) absorbing sunscreen synthesis (genes for mycosporine-like amino acids and scytonemin biosynthesis). Our comparative genome analysis of these two *Leptolyngbya* strains provides insights into the adaptation strategies that these strains use to thrive under terrestrial and freshwater conditions.

## 2. Materials and methods

### 2.1. Genome sequencing and assembly

*Leptolyngbya* sp. NIES-2104, a strain isolated from crusts of another terrestrial cyanobacterium, *N. commune* HK-02 (NIES-2114)^12^ at Himeji, Hyogo, Japan, 2002, was subsequently acquired by the Microbial Culture Collection (MCC) of the National Institute for Environmental Studies (NIES), Japan (http://mcc.nies.go.jp). Purity of the culture was assayed as previously described.^21^ Genomic DNA of *Leptolyngbya* sp. NIES-2104 was extracted by bead beating and then was purified using a DNeasy Plant Mini Kit (QIAGEN, Venlo, the Netherlands). A paired-end library was prepared using a TruSeq DNA PCR-Free Sample Prep Kit (Illumina, San Diego, CA, USA) after fragmentation with a Covaris M220 (Covaris, Woburn, MA, USA). A mate-pair library of 8 kb inserts was prepared using the ‘gel-plus’ protocol of the Nextera Mate-Pair Sample Prep Kit (Illumina). Both libraries were sequenced with MiSeq Reagent Kit v3 600 cycles (Illumina) on a MiSeq instrument. We obtained 2,447,760 pair-end reads and 812,724 mate-pair reads as the output of MiSeq. Then, reads were *de novo* assembled using Newbler v2.9 (Roche Applied Science, Penzberg, Germany), which yielded 61 contigs and 8 scaffolds. Sequences of gaps between contigs and scaffolds were determined *in silico* using two programs—GenoFinisher and Ace File Viewer (both distributed by Dr Y. Ohtsubo of Tohoku University, Japan).^22^ Finally, gap-less sequences of five contigs (most likely one chromosome and four plasmids) were obtained. The complete genome sequence of *Leptolyngbya* sp. NIES-2104 was deposited in the DDBJ database (http://www.ddbj.nig.ac.jp) with the accession numbers BBWW01000001–BBWW01000005.

### 2.2. Phylogenetic analysis

For molecular phylogenetic analyses, 16S rDNA sequences (except for that of *Leptolyngbya* sp. NIES-2104) were retrieved from GenBank (https://www.ncbi.nlm.nih.gov/genbank/). We performed a phylogenetic analysis of *Leptolyngbya* sp. NIES-2104 with 16S rDNA sequences for 98 strains assigned as *Leptolyngbya* and 16S rDNA sequences of 4 strains and three uncultured cyanobacterium clones that showed high similarity to the 16S rDNA sequence of *Leptolyngbya* sp. NIES-2104 or other *Leptolyngbya* strains. The 16S rDNA sequences of *Gloeobacter violaceus* sp. PCC 7421 and *Bacillus subtilis* subsp. *subtilis* strain 168 were used as outgroups. The GenBank accession numbers for each OTU are listed in Fig. 1. Multiple sequence alignment was conducted using the MUSCLE algorithm implemented in MEGA5.2.2.^23,24^ A phylogenetic tree was constructed using maximum-likelihood (ML) algorithms based on the K2+G+I model with 1000 bootstrap replications using the MEGA 5.2.2 package.^23,24^

### 2.3. Genome annotation of *Leptolyngbya* sp. NIES-2104 and comparison with the genomes of *Leptolyngbya boryana* PCC 6306 and *Nostoc* sp. PCC 7120

The genome sequence of *Leptolyngbya* sp. NIES-2104 and the draft genome sequence of *L. boryana* PCC 6306 retrieved from GenBank (accession numbers: KB731324, KB731325, KB731326, KB731327, and KB731328) were submitted to RAST^25^ using similar settings. Bidirectional blast best-hit pairs (query coverage ≥90%, percentage of identical matches ≥50) of the deduced proteins were considered orthologous. Deduced protein sequences of *Nostoc* sp. PCC 7120 were also retrieved from GenBank (accession numbers: NC_003272, NC_003276, NC_003240, NC_003267, NC_003273, NC_003270, and NC_003241) and were compared with those of *Leptolyngbya* sp. NIES-2104 and *L. boryana* PCC 6306.

### 2.4. Synteny analysis

Whole genome synteny between *Leptolyngbya* sp. NIES-2104 and *L. boryana* PCC6306 was analysed using MURASAKI^26^ (with a 25-bp seed that allows for a mismatch at 7 specific points: 1111110011110011111000111) and was visualized using nmny (http://mux.eee.u-ryukyu.ac.jp/nmny/trial).

Syntenies of the *nif* gene cluster (nitrogen fixation-related genes), the micro-oxic condition-inducible tetrapyrrole biosynthetic gene cluster, the *tre* gene cluster (genes for trehalose metabolism), the mycosporine-like amino acid synthesis gene cluster, and the flanking regions between cyanobacterial strains were analysed and visualized using a program for comparing genome sequences, GenomeMatcher.^22^ GenBank accession numbers of the sequences and the loci analysed are described in Figs 3–6 and in the corresponding figure legends.

## 3. Results and discussion

### 3.1. Phylogenetic positioning of *Leptolyngbya* sp. NIES-2104 within the phylum cyanobacteria and comparisons with other *Leptolyngbya* strains

There are notable examples that morphologically similar cyanobacteria occupy widely divergent positions within the molecular phylogenetic tree of cyanobacteria, although they are currently assigned to the same genus.^10^ This situation is controversial and problematic for cyanobacterial taxonomy. The form genus *Leptolyngbya*^9^ is also polyphyletic in the 16S rDNA sequence-based phylogenetic tree.^10^ Accordingly, the phylogenetic position must be investigated for each individual strain. To investigate the phylogenetic position of *Leptolyngbya* sp. NIES-2104, we performed a phylogenetic analysis based on 16S rDNA sequences (Fig. 1).

From our analysis, some *Leptolyngbya* strains formed a clade with high bootstrap support (Clade A–G, Fig. 1). *Leptolyngbya* sp. NIES-2104 clustered with *Leptolyngbya sensu stricto* (clade A-2, Fig. 1) in a clade that includes type species *L. boryana* (e.g. *L. boryana* PCC 6306, which is a reference strain for Cluster 1 of the form genus *Leptolyngbya*,^10^ and Cluster 1 corresponds to Clade A-2 in Fig. 1). Similarities among 16S rDNA sequences, which are included in Clade A (Fig. 1), are shown in Supplementary Table S1. Among Clade A, the 16S rDNA sequence of *Leptolyngbya* sp. NIES-2104 was most similar (96.7%) to that of *Leptolyngbya* sp. SEV4-3-C1 (AY239596). All SEV strains shown in Fig. 1 and Supplementary Table S1 had been isolated from desert soils,^2^ and *Leptolyngbya* sp. NIES-2104 had also been isolated from a terrestrial environment and is desiccation tolerant. Therefore, Clade A-2 (Fig. 1) is thought to be a terrestrial *Leptolyngbya* clade. Clade A also contains the recently proposed genus *Plectolyngbya*;^27^ however, the phylogenetic relationship of this genus with Clades A-1 and A-2 was unclear in our analysis.

In Clade A (Fig. 1), the genome of *L. boryana* PCC 6306 had previously been sequenced.^4^ The identity of the 16S rDNA sequences between *Leptolyngbya* sp. NIES-2104 and *L. boryana* PCC 6306 is ∼95.3% (Supplementary Table S1); this value is slightly above the threshold of distinction for certain cyanobacterial genera (95%).^28,29^ Generally, bacterial strains that have a 16S rDNA sequence identity <97.5% are not likely to show DNA–DNA hybridization values >70% and are referred to as distinct species.^30^ *Leptolyngbya* sp. NIES-2104 does not show 16S rDNA sequence identity >97.5% with any of the sequenced organisms. Hence, *Leptolyngbya* sp. NIES-2104 is most clearly related to the genus *Leptolyngbya sensu stricto* (Clade A-2, Fig. 1) and is probably a novel species.

### 3.2. General properties of the *Leptolyngbya* sp. NIES-2104 genome and a comparison with those of *Leptolyngbya boryana* PCC 6306

*Leptolyngbya* sp. NIES-2104 is a non-nitrogen-fixing terrestrial strain, whereas *L. boryana* PCC 6306 is nitrogen-fixing freshwater strain. Herein, we compared the genome sequence of *Leptolyngbya* sp. NIES-2104 with that of *L. boryana* PCC 6306 and inferred the genetic factors that contribute to habitat differentiation.

While the genome sequence of *L. boryana* PCC 6306 is nearly completed (5 scaffolds comprise 19 contigs), we successfully obtained a complete genome sequence of *Leptolyngbya* sp. NIES-2104 (DDBJ accession numbers: BBWW01000001–BBWW01000005). General information about the genome is presented in Table 1. The genome of *Leptolyngbya* sp. NIES-2104 is composed of five circular DNA molecules (most likely one circular chromosome and four plasmids). The size of the largest circular DNA is 5,695,116 bp, and it contains 5,961 CDSs and all predicted RNA genes. The sizes of the other four DNAs are 318,180, 263,280, 84,246, and 25,488 bp, and these contain 329, 315, 81, and 26 CDSs, respectively. The total size of the *Leptolyngbya* sp. NIES-2104 genome is 6,386,310 bp, which is smaller than that of the 7,261,054 bp of *L. boryana* PCC 6306 genome. The GC content of the genome is 47.4%, which is comparable to the 47.0% of *L. boryana* PCC6306. There are three rDNA operons in the *Leptolyngbya* sp. NIES-2104 genome, which is similar to the *L. boryana* PCC 6306 genome. Some cyanobacteria have rDNA operon variants in their genome, such as *Nostoc* sp. PCC7120.^31^ Some strains of *L. boryana* (UTEX B 487, UTEX B 485, UTEX B 482, and UTEX B 488) have rDNA operon variants (the major difference between the variants is the presence or absence of both genes for tRNA^Ile^ and tRNA^Ala^ at the 16S-23S internal transcribed spacer region) in the genome, and the SEV strains do not have those variants.^32^ As predicted from our phylogenetic analysis (Fig. 1), *Leptolyngbya* sp. NIES-2104 appears to lack the variant in the rDNA operon in its genome, as do the SEV strains. However, in contrast to our expectations, we could not identify the variant in the rDNA operons in the genome of *L. boryana* PCC 6306 (GenBank accession number: KB731324–KB731328), although strain PCC 6306 is virtually identical to strain UTEX B 482.^4^ This unexpected finding is presumably a consequence of the short-read sequencing and alignment used to assemble the genome of strain PCC 6306.

**Table 1.** General information about the genomes of two *Leptolyngbya* strains

	*Leptolyngbya* sp. NIES-2104	*Leptolyngbya boryana* PCC 6306
Genome size (bp)	6,386,307	7,261,054
Number of contigs	5	19
GC content (%)	47.4	47.0
Number of rDNA operons	3	3
Number of rRNA + tRNA genes	60	73
Number of coding sequences	6,712	7,355

Note: The genome of *Leptolyngyba* sp. NIES-2104 does not contain any gaps, whereas the genome of *L. boryana* PCC 6306 contains 14 gaps.

The RAST annotation pipeline^25^ predicted 6,712 and 7,355 coding sequences for the *Leptolyngbya* sp. NIES-2104 and *L. boryana* PCC 6306 genomes, respectively, although these numbers of coding sequences are overestimates, because the originally published *L. boryana* PCC 6306 genome contains only 5,944 protein coding sequences (NCBI reference sequence accession number: NZ_ALVM00000000). Among those coding sequences predicted by RAST, we estimate that 3,378 protein pairs are orthologous based on a BLAST bi-directional best-hit strategy, so approximately half of the predicted protein sequences of *Leptolyngbya* sp. NIES-2014 have a high similarity value to *L. boryana* PCC 6306 orthologs. These orthologous proteins might define the physiological similarity of Clade A (Fig. 1), whereas the others could serve as the basis for the physiological differences. When similar approaches for predicting orthologous protein pairs were used against the filamentous nitrogen-fixing cyanobacterium *Nostoc* sp. PCC 7120 genome sequence, the numbers of ‘hits’ returned were 1,860 (*Leptolyngbya* sp. NIES-2104 vs. *Nostoc* sp. PCC 7120) and 1,954 (*L. boryana* PCC 6306 vs. *Nostoc* sp. PCC 7120) orthologous protein pairs. These numbers represent a reference for the number of proteins that are conserved among cyanobacteria, even between species that are not closely related.

**Figure 2.**
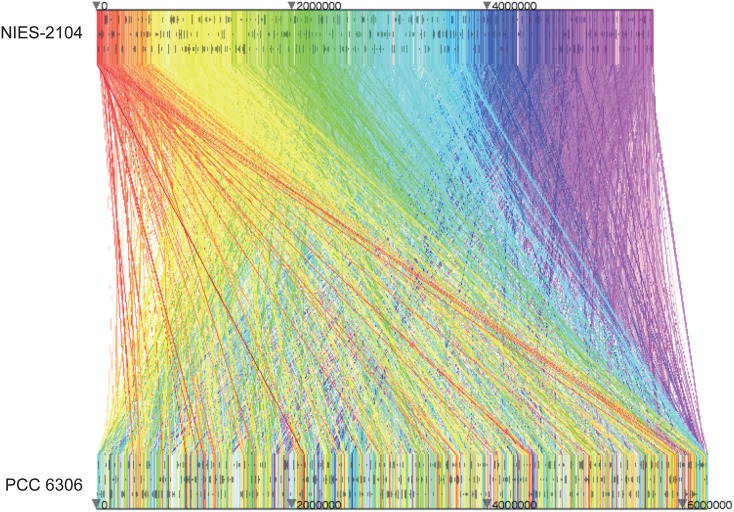
Genomic synteny between *Leptolyngbya* sp. NIES-2104 and *Leptolyngbya boryana* PCC 6306. The regions that show similarity between these two genomes are connected by colored lines. The genome sequence at the top is *Leptolyngbya* sp. NIES-2104 and the sequence at the bottom is *L. boryana* PCC 6306.

**Figure 3.**
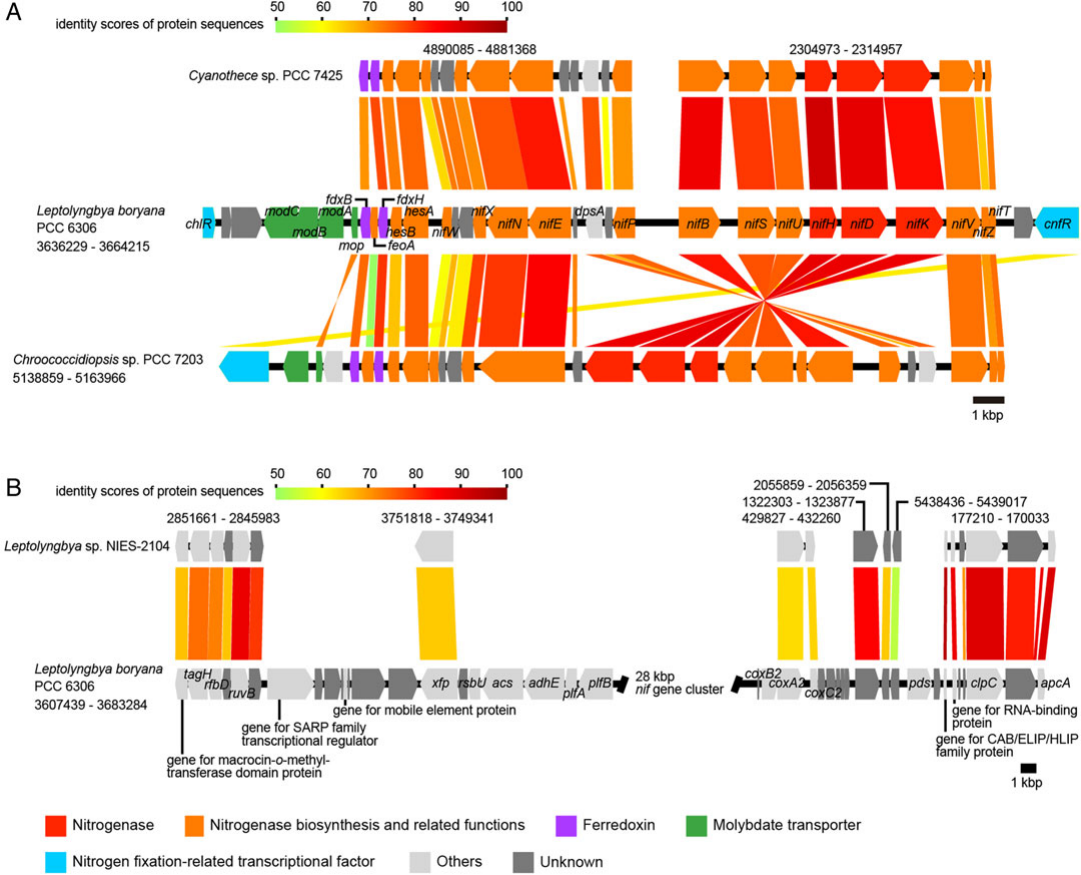
Synteny analysis of the *nif* gene cluster and its flanking regions in *Leptolyngbya boryana* PCC 6306. A comparison of *nif* gene clusters from three cyanobacterial strains (A). A comparison of the flanking region of the *nif* gene cluster in *Leptolyngbya boryana* PCC 6306 with the genome of *Leptolyngbya* sp. NIES-2104 (B). GenBank accession numbers are as follows: KB731324 for *L. boryana* PCC 6306, CP001344 for *Cyanothece* sp. PCC 7425, CP003597 for *Chroococcidiopsis thermalis* PCC 7203, and DDBJ accession number for *Leptolyngbya* sp. NIES-2104 is BBWW01000001. Loci are indicated by numbers at both ends. All genes are color-coded based on function, as shown in the lower panel. Homologous genes are connected by color lines, and these colors indicate the identity score of protein sequences, which are shown in the upper part of each panel.

**Figure 4.**
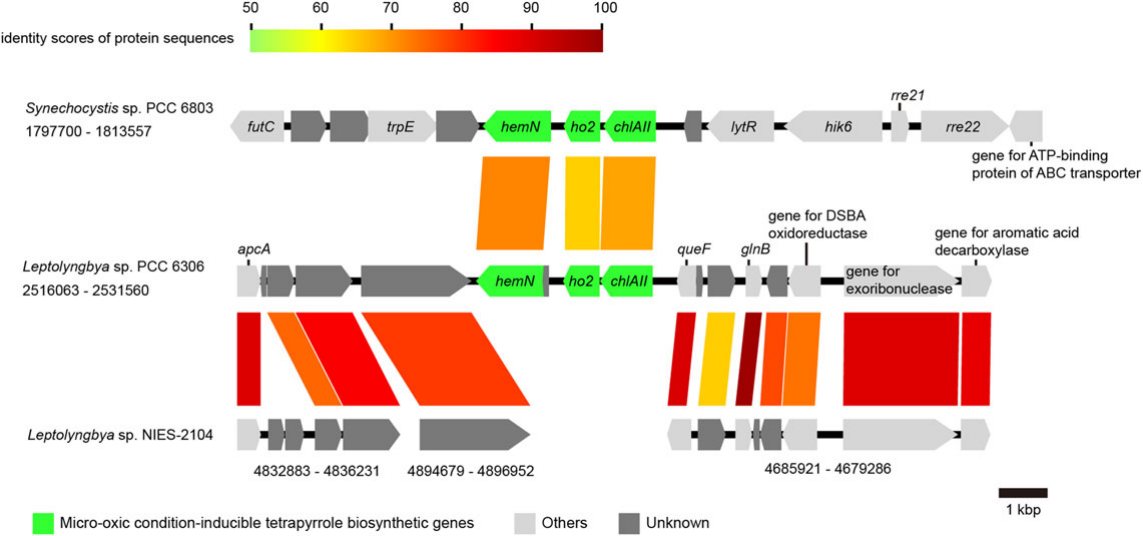
A comparison of the micro-oxic condition-inducible tetrapyrrole biosynthetic genes and its flanking regions in cyanobacteria. GenBank accession numbers are as follows: BA000022.2 for *Synechocystis* sp. PCC 6803, KB731324 for *Leptolyngbya boryana* PCC 6306, and DDBJ accession number for *Leptolyngbya* sp. NIES-2104 is BBWW01000001. Loci are indicated by numbers at both ends. All genes are color-coded based on function, as shown in the lower panel. Homologous genes are connected by colored lines, and colors indicate the identity score of protein sequences, as shown in the upper part of the panel.

**Figure 5.**
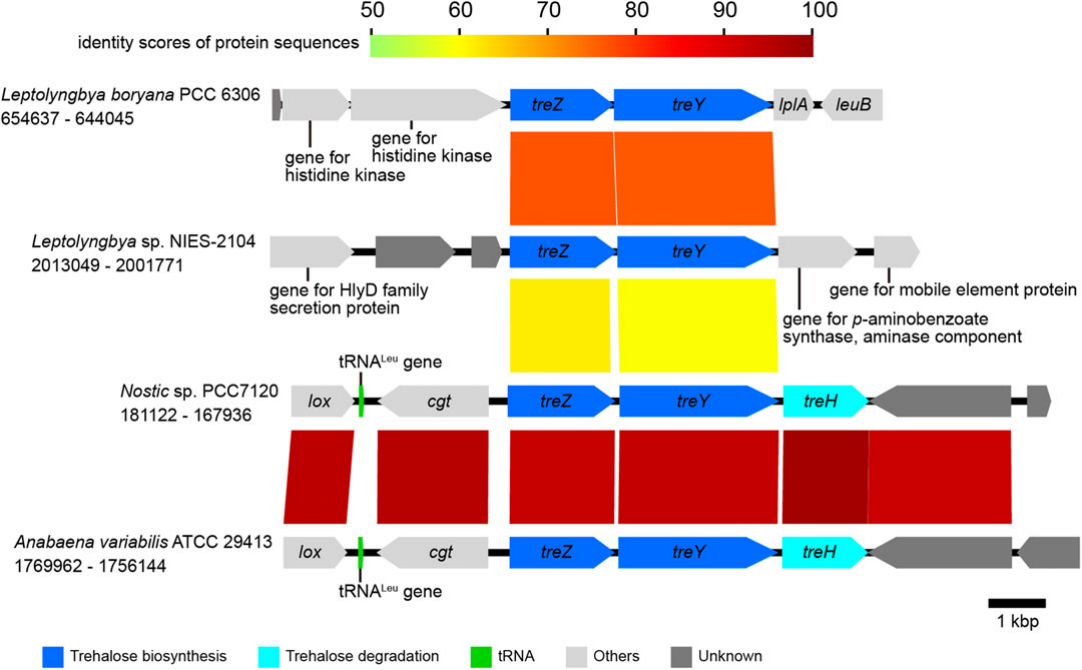
A comparison of the *tre* gene cluster and its flanking regions in cyanobacteria. GenBank accession numbers are as follows: KB731324 for *Leptolyngbya boryana* PCC 6306, NC_003272 for *Nostoc* sp. PCC 7120, CP000117 for *Anabaena variabilis* ATCC 29413, and DDBJ accession number for *Leptolyngbya* sp. NIES-2104 is BBWW01000001. Loci are indicated by numbers at both ends. All genes are color-coded based on function, as shown in the lower panel. Homologous genes are connected by colored lines, and colors indicate the identity score of protein sequences, as shown in the upper part of the panel.

**Figure 6.**
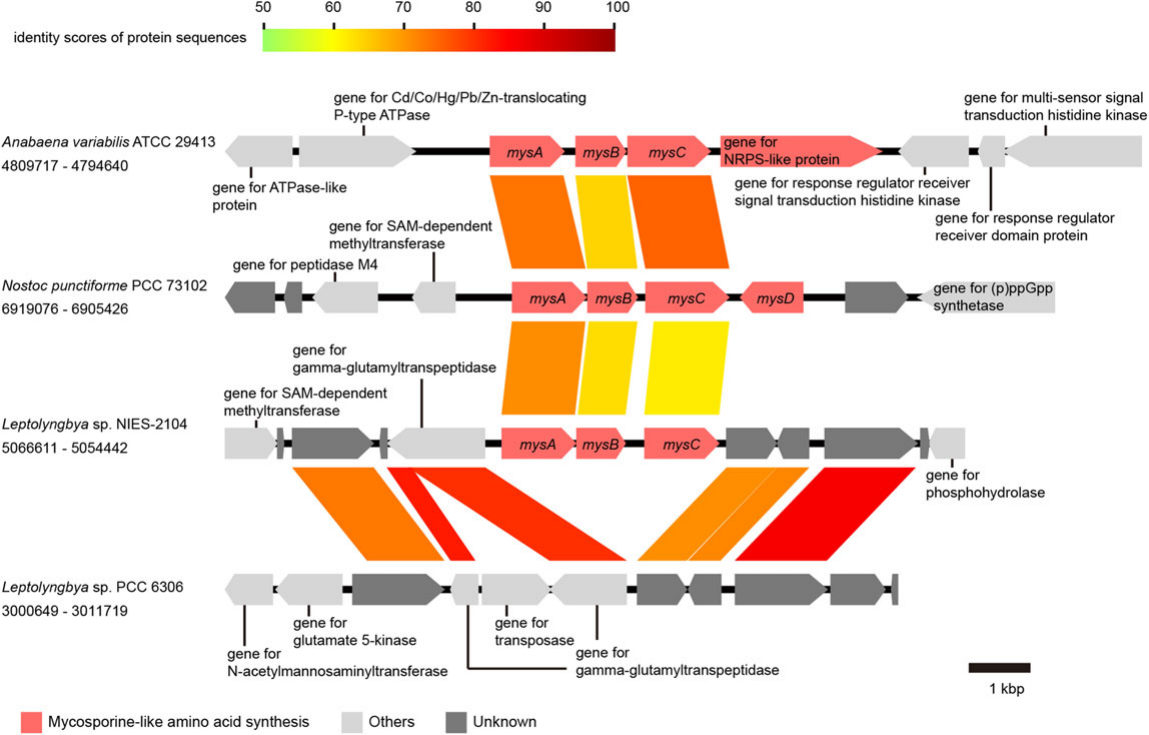
A comparison of the gene cluster for the synthesis of mycosporine-like amino acids and its flanking regions in cyanobacteria. GenBank accession numbers are as follows: CP000117 for *Anabaena variabilis* ATCC 29413, CP001037 for *Nostoc punctiforme* PCC 73102, KB731324 for *Leptolyngbya boryana* PCC 6306, and DDBJ accession number for *Leptolyngbya* sp. NIES-2104 is BBWW01000001. Loci are indicated by numbers at both ends. All genes are color-coded based on function, as shown in the lower panel. Homologous genes are connected by colored lines, and colors indicate the identity score of protein sequences, as shown in the upper part of the panel.

Genome-wide synteny between *Leptolyngbya* sp. NIES-2104 and *L. boryana* PCC 6306 was analysed using MURASAKI, and the results were visualized using nmny (Fig. 2). Small-scale synteny was somewhat conserved (see also Figs 3B, 4–6), but large-scale synteny was not observed between these two genomes (Fig. 2).

### 3.3. Nitrogen fixation-related genes in *Leptolyngbya* sp. NIES-2104 and *Leptolyngbya boryana* PCC 6306

Some filamentous cyanobacteria develop specialized cells, termed heterocysts, for nitrogen fixation within the trichome under nitrogen starvation conditions, and heterocyst provide micro-oxic conditions for oxygen-labile nitrogen-fixing enzyme, nitrogenase. Alternatively, nitrogen fixation by non-heterocystous cyanobacteria has been reported in many genera,^33^ e.g. *Leptolyngbya* (formerly known as *Plectonema*), *Gloeothece*, and *Chroococcidiopsis*. Within the authentic *Leptolyngbya* clade (Clade A, Fig. 1), many strains, such as *L. boryana* IAM M-101, *L. boryana* PCC 6306, and *Leptolyngbya* sp. PCC 73110, fix nitrogen under anaerobic conditions^1,34^ (note that *L. boryana* strain *dg5* is a dark-adapted strain derived from strain IAM M-101^18,34^). The genome sequence of *L. boryana* sp. PCC 6306 contains a large gene cluster for nitrogen fixation, which is originally identified in the strain *dg5*.^34^ A BLAST search of the nitrogen fixation-related proteins from the *L. boryana* PCC 6306 genome against *Leptolyngbya* sp. NIES-2104 did not return any significant similarities. We also searched for pseudogenes of nitrogen fixation-related genes in *Leptolyngbya* sp. NIES-2104 using the DNA sequences of nitrogen fixation-related genes of *L. boryana* PCC 6306 as a query, but could not detect any similar sequences.

When analysing synteny of the *nif* gene cluster of *L. boryana* PCC 6306 compared with other cyanobacteria, ∼28 kb of the *nif* gene cluster of *L. boryana* PCC 6306 was highly similar to those of non-heterocystous cyanobacteria, such as *Chroococcidiopsis thermalis* PCC 7203 and *Cyanothece* sp. PCC 7425. We note that the cluster of the *Cyanothece* could be split into two genomic loci and the cluster of the *Chroococcidiopsis* contained an inversion within the locus (Fig. 3A). A ∼63-kb genomic region of *L. boryana* PCC 6306, which contains a 28-kbp *nif* gene cluster, was missing in the *Leptolyngbya* sp. NIES-2104 genome (Fig. 3B). Homologous genes found in the flanking regions were dispersed in the *Leptolyngbya* sp. NIES-2104 genome, probably as a consequence of numerous genome rearrangements (Fig. 3B). This case is very similar to the differences that were observed within another cyanobacterial genus, *Acaryochloris*.^35^ Horizontal gene transfer of the *nif* gene cluster from another bacterial phylum to a cyanobacterial lineage has been reported;^36^ however, *nif* genes in the *L. boryana* PCC 6306 genome have high similarity to those of other cyanobacteria (Fig. 3A). This finding indicates that the *nif* gene cluster of *L. boryana* was inherited from its ancestor or was laterally transferred from another cyanobacterial lineage, and at least the *nif* gene clusters of *L. boryana* and *C. thermalis* PCC 7203 appear to share a common origin. In this study, we could not elucidate whether *Leptolyngbya* sp. NIES-2104 had lost its nitrogen fixation gene cluster or whether *L. boryana* PCC 6306 had acquired its nitrogen fixation gene cluster after the bifurcation of its ancestors. Further studies to investigate the distribution of the nitrogen fixation gene cluster within the authentic *Leptolyngbya* clade (Clade A, Fig. 1), and the genomic features of strains that map near the base of the clade will be needed to answer this question. *Leptolyngbya* sp. NIES-2104 was isolated from a colony of *N. commune* that inhabited the soil surface. *Nostoc commune* is a heterocystous cyanobacterium that can fix nitrogen, even under aerobic conditions. Terrestrial non-nitrogen-fixing *Leptolyngbya* can inhabit areas where available forms of nitrogen are replete, and multiple sources of nitrogen might be supplied by cohabiting nitrogen-fixing microorganisms, such as heterocystous cyanobacteria.

Nitrogenase is an oxygen-labile protein, and recently the expression of the gene encoding nitrogenase in *L. boryana* was found to be regulated by the CnfR (cyanobacterial nitrogen fixation regulator) protein.^34^ CnfR is expressed under nitrogen-starvation conditions and can be activated under micro-oxic conditions.^34^ Gas diffusion is very limited in water compared with soil surfaces. Oxygen levels can be depressed by the respiration of microorganisms, and micro-oxic conditions frequently occur at the bottom of bodies of water. By contrast, micro-oxic conditions, which are needed for the expression and activity of nitrogenase, might rarely occur on the soil surface where there is a higher rate of gas diffusion, and nitrogen fixation by non-heterocystous cyanobacteria might be much more difficult on the soil surface than in water bodies. The difference in gas diffusion rates between these habitats could contribute the presence or absence of genes for nitrogen fixation in the species that we analysed.

Regarding biological nitrogen fixation, nitrogenase also produces molecular hydrogen (H_2_) as a side product of ammonia.^37^ Most nitrogen-fixing cyanobacteria have ‘uptake hydrogenase’ for the oxidation of molecular hydrogen and the reproduction of a reductant.^37,38^ However, *L. boryana* PCC 6306 does not encode any homologous gene for uptake hydrogenase and neither does *Leptolyngbya* sp. NIES-2104. The genome sequences of these two organisms do not encode homologous genes for any known hydrogenase enzyme (neither uptake hydrogenase nor bi-directional hydrogenase). The lack of a hydrogenase enzyme might be one of the common genomic features of these two *Leptolyngbya* lineages (Clades A-1 and A-2, Fig. 1).

### 3.4. Micro-oxic condition-inducible tetrapyrrole biosynthetic genes

The oxygen levels in environments where cyanobacteria live can be highly variable, and in some closed environments, cyanobacteria are often exposed to micro-oxic conditions because of the respiration of other bacteria and self-respiration.^39^ In such situations, cyanobacteria use some analogous enzymes for tetrapyrrole biosynthesis, which mainly operate under micro-oxic conditions, along with enzymes that mainly operate under aerobic conditions. Among those enzymes, the genes that encode ChlA_II_ (Mg-protoporphyrin IX monomethylester cyclase),^40^ HO2 (heme oxygenase),^41^ and HemN (oxygen-independent coproporphyrinogen III oxidase)^42^ are inducible under micro-oxic conditions, and their expression can be regulated by the MarR-type transcriptional regulator ChlR.^43^ ChlR is constitutively expressed and exerts positive transcriptional regulation activity under micro-oxic conditions.^34^ These genes and their regulation are conserved in *L. boryana dg5*,^34^ and we could confirm the existence of these genes within the PCC 6306 genome. However, the *Leptolyngbya* sp. NIES-2104 genome does not encode any of these genes (Fig. 4).

*Leptolyngbya* sp. NIES-2104 may have lost the micro-oxic inducible genes as a consequence of adaptations to more oxidative conditions.

### 3.5. Trehalose-related metabolism of *Leptolyngbya* sp. NIES-2104

Water availability is one of the limiting factors that define the habitat of a microorganism. Compared with aquatic species, terrestrial species are water challenged and must undergo desiccation and rehydration cycles more often. To overcome these environmental stresses, microorganisms use non-reducing sugars, such as sucrose and trehalose. Trehalose is thought to extend desiccation tolerance for many types of organisms,^44,45^ including cyanobacteria,^46^ and trehalose content may be one of the factors that defines desiccation tolerance in bacteria. Desiccation-tolerant strains of cyanobacteria accumulate high amounts of trehalose and/or sucrose compared with desiccation-sensitive strains.^46,47^ *L. boryana* (formerly *Plectonema boryanum*) has been reported to be desiccation intolerant and accumulate a very small amount of sucrose under desiccation stress conditions.^46^

In cyanobacteria, trehalose is synthesized from glycogen via α-1,1-maltooligosyltrehalose, a reaction that can be catalyzed by maltooligosyl trehalose synthase (EC 5.4.99.15), which is encoded by the *treY* gene, and maltooligosyl trehalose trehalohydrolase (EC 3.2.1.141), which is encoded by the *treZ* gene.^48^ In a genomic survey of trehalose-related metabolic genes in the *Leptolyngbya* sp. NIES-2104 genome, we could identify a *treZY* gene cluster; we also found a similar cluster in the *L. boryana* PCC 6306 genome (Fig. 5). In *Nostoc* species, *treZY* genes were followed by the *treH* gene for α,α-trehalase (EC 3.2.1.28), which hydrolyzes a trehalose to two glucoses^47,49^ (Fig. 5). The organization of *treZYH* is conserved between heterocystous cyanobacteria, *Nostoc*, and *Anabaena* species (Fig. 5). However, we could not identify such organization or any homologous genes for *treH* in the *Leptolyngbya* sp. NIES-2104 or *L. boryana* PCC 6306 genomes. *Scytonema* species, which were isolated from desert soil, have been reported to lack α,α-trehalase (EC 3.2.1.28) and phosphotrehalase (EC 3.2.1.93) activities and only possesses trehalose phosphorylase (EC 2.4.1.64) activity.^50^ We performed BLAST searches for trehalose phosphorylase against the RAST annotated genome sequences of *Leptolyngbya* sp. NIES-2104 and *L. boryana* PCC 6306 using the enzymologically characterized trehalose phosphorylase sequence of *Thermoanaerobacter brockii* ATCC 35047 (GenBank accession number: BAB97299)^51,52^ as a query. Then, three deduced protein sequences in each *Leptolyngbya* strain were found to have high similarity with trehalose phosphorylase of *T. brockii* ATCC 35047 (Table 2). Trehalose might be degraded by these putative trehalose phosphorylases in these *Leptolyngbya* strains. In addition to the *treZY* trehalose synthetic gene cluster, we identified *treS* gene homologues for trehalose synthase (EC 5.4.99.16) in both the *Leptolyngbya* sp. NIES-2104 and *L. boryana* PCC 6306 genomes. TreS can convert maltose into trehalose, or trehalose into maltose, by intramolecular transglucosylation.^53,54^ In some bacteria, the flux through TreS flows from trehalose to maltose, and the over-expression of TreS causes reductions in cellular trehalose content.^55^ The function of the protein encoded by the *treS* homologue is not known in cyanobacteria, and the deduced TreS protein sequences in the two *Leptolyngbya* strains include a long C-terminal extension compared with TreS of *Mycobacterium tuberculosis* H37Rv (NCBI reference sequence accession number: NP_214640). Enzymatic characterization of the *treS* gene product will be needed to better understand trehalose-related metabolism in these cyanobacteria.

**Table 2.** Trehalose phosphorylase enzymes in two *Leptolyngbya* strains

	Length, amino acid residues	Identities (%)	Positives (%)	Gaps (%)
*Leptolyngbya boryana* PCC 6306				
NCBI reference sequence accession number				
WP_017291470	787	42	61	1
WP_017290423	750	31	51	7
WP_017290913	968	32	51	9
*Leptolyngbya* sp. NIES-2104				
Locus_tag in *Leptolyngbya* sp. NIES-2104 genome				
NIES2104_14780	801	42	62	1
NIES2104_03880	745	33	51	7
NIES2104_30690	965	33	52	8

Identities, positives, and gaps represent the results of a BLAST search, in which trehalose phosphorylase from *Thermoanaerobacter brockii* ATCC35047 (BAB97299) was used as a query.

Herein, we found evidence that both aquatic and terrestrial strains (PCC 6306 and NIES-2104) have the genetic capacity to produce trehalose. However, the amount of trehalose that can accumulate in cells of these two strains under desiccation conditions remains unknown. The trehalose content in these strains or regulation of the expression of genes for trehalose synthesis or degradation might be different, and such differences might contribute to differences in desiccation tolerance. Additional physiological studies will be required to test these hypotheses. For *Leptolyngbya* sp. NIES-2104, this strain was isolated from a colony of *N. commune*, so its desiccation tolerance might be enhanced *in situ* by extracellular polysaccharides derived from the cohabitant *N. commune*. Indeed, it has been reported that extracellular polysaccharides of terrestrial desiccation-tolerant *Nostoc* species have important roles in desiccation tolerance, not only for itself, but probably for its cohabitants as well.^56,57^

### 3.6. Genes for UV absorbing sunscreen synthesis

On soil surfaces, microorganisms are challenged by higher amounts of radiation from solar light than in most other environments. Phototrophic organisms use sunlight as an energy source, but are also exposed to harmful UV radiation. To prevent the deleterious effects of UV radiation, they synthesize UV-absorbing sunscreens.^58^ Mycosporine-like amino acids are UV-absorbing small molecules that are found in many cyanobacterial lineages.^59^ In cyanobacteria, two types of conserved gene clusters for the synthesis of a mycosporine-like amino acid, shinorine, have been characterized.^60,61^ These gene clusters each include four genes and share three genes that catalyze the reaction from sedoheptulose 7-phosphate to mycosporine-glycine, *mysA* (a gene that encodes a 2-demethyl-4-deoxygadusol synthase that acts on sedoheptulose 7-phosphate), *mysB* (a gene that encodes a *O*-methyltransferase that catalyzes the methylation of 2-demethyl-4-deoxygadusol, yielding the common core of mycosporine-like amino acids, 4-deoxygadusol), and *mysC* (a gene that encodes the protein that catalyzes the condensation of glycine to 4-deoxygadusol). The final step for the synthesis of shinorine differs between the two types, as one is catalyzed by the non-ribosomal peptide synthase (NRPS)-like protein, and another is catalyzed by the ATP-grasp ligase, which is encoded by the *mysD* gene. The mycosporine synthesis gene cluster could be found in *Leptolyngbya* sp. NIES-2104, but not in *L. boryana* PCC 6306 (Fig. 6). The mycosporine synthesis gene cluster of *Leptolyngbya* sp. NIES-2104 contains only *mysABC*, and neither *mysD* nor the gene for the NRPS-like protein homologue for biosynthesis of shinorine exists in the genome of *Leptolyngbya* sp. NIES-2104 (Fig. 6). *Leptolyngbya* sp. NIES-2104 clearly exhibits the genetic capacity to produce mycosporine-glycine. This product might contribute to defence against UV radiation under terrestrial conditions. Furthermore, mycosporine-like amino acids, especially mycosporine-glycine, works both as a sunscreen and as an antioxidant.^62^ High light irradiation and salt stress accompanied by desiccation can lead to oxidative stresses.^63^ Mycosporine-glycine might increase fitness under terrestrial conditions by working both as a sunscreen and as an antioxidant.

Some cyanobacteria can also synthesize another type of sunscreen, scytonemin^58^; however, genes for scytonemin synthesis were not identified in either *Leptolyngbya* strain (NIES-2014 and PCC 6306).

### 3.7. Future prospects

Herein, we discussed the genetic features of *L. boryana* PCC 6306 and *Leptolyngbya* sp. NIES-2104 in light of their different habitats. *Leptolyngbya* is a simple filamentous cyanobacterial genus, and the morphological differences between its species are subtle. The lowest similarity of 16S rDNA sequences among the authentic strains is 91.2% between *Leptolyngbya* sp. SEV5-3-C28 and *Leptolyngbya* cf. Albertano–Kovacik green Es Yyy1800 (Supplementary Table S1). This value seems to be too low to support all of the strains included in the clade as genus *Leptolyngbya*. Although the currently available sequence data and ecological records for this clade are limited, we can now initiate physiological experiments based on the insights yielded by these genomic features. Physiological differences that result from genomic differences could aid in the classification of this clade and provide more credible evidence for ecological differentiation.

## Supplementary data

Supplementary data are available at www.dnaresearch.oxfordjournals.org.

## Funding

Funding to pay the Open Access publication charges for this article was provided by the Japan Agency for Medical Research and Development (AMED), and the Ministry of Education, Culture, Sports, Science, and Technology of Japan.

## Supplementary Material

Supplementary Data
