# Supplementary material for: Comparison of the terrestrial cyanobacterium Leptolyngbya sp. NIES-2104 and the freshwater Leptolyngbya boryana PCC 6306 genomes
Source: DNA Res. 2015 Oct 21;22(6):403–12. doi: 10.1093/dnares/dsv022 (PMC4675709; doi:10.1093/dnares/dsv022)
Supplement: Supplementary Data [file supp_22_6_403__index.html]

Comparison of the terrestrial cyanobacterium Leptolyngbya sp. NIES-2104 and the freshwater Leptolyngbya boryana PCC 6306 genomes — Supplementary Data 

# Comparison of the terrestrial cyanobacterium *Leptolyngbya* sp. NIES-2104 and the freshwater *Leptolyngbya boryana* PCC 6306 genomes

## Supplementary Data

Supplementary Data

- Supplementary Table 1 - xls file
